# Evaluation and Comparison of Serological Methods for COVID-19 Diagnosis

**DOI:** 10.3389/fmolb.2021.682405

**Published:** 2021-07-23

**Authors:** Fanwu Gong, Hua-xing Wei, Qiangsheng Li, Liu Liu, Bofeng Li

**Affiliations:** ^1^ Department of Medical Oncology, The First Affiliated Hospital of USTC, Division of Life Sciences and Medicine, University of Science and Technology of China, Hefei, China; ^2^ Department of Laboratory Medicine, The First Affiliated Hospital of USTC, Division of Life Sciences and Medicine, University of Science and Technology of China, Hefei, China; ^3^ Department of General Surgery, The First Affiliated Hospital of USTC, University of Science and Technology of China, Hefei, China

**Keywords:** COVID-19 diagnosis, serological testing, antibody, SARS-CoV-2, ELISA, LFIA

## Abstract

The worldwide pandemic of COVID-19 has become a global public health crisis. Various clinical diagnosis methods have been developed to distinguish COVID-19–infected patients from healthy people. The nucleic acid test is the golden standard for virus detection as it is suitable for early diagnosis. However, due to the low amount of viral nucleic acid in the respiratory tract, the sensitivity of nucleic acid detection is unsatisfactory. As a result, serological screening began to be widely used with the merits of simple procedures, lower cost, and shorter detection time. Serological tests currently include the enzyme-linked immunosorbent assay (ELISA), lateral flow immunoassay (LFIA), and chemiluminescence immunoassay (CLIA). This review describes various serological methods, discusses the performance and diagnostic effects of different methods, and points out the problems and the direction of optimization, to improve the efficiency of clinical diagnosis. These increasingly sophisticated and diverse serological diagnostic technologies will help human beings to control the spread of COVID-19.

## Introduction

The global public health and economy have steadily deteriorated by the spread of SARS-CoV-2 (severe acute respiratory syndrome coronavirus 2) (Asselah et al., 2021). Up to now, SARS-CoV-2 has infected more than 119 million people, resulting in more than 2,600,000 deaths. On December 8, 2019, the first SARS-CoV-2 infection case was reported in Wuhan, China. The Chinese government announced the existence of the novel coronavirus and took effective measures to control the spread of the virus (The Lancet 2020; Zhang et al., 2020). Unfortunately, the virus spread rapidly and widely all over the world (Krouse, 2020; Krishnan et al., 2021). Interestingly, a recent study reported 106 out of 7,389 blood samples, with an earliest donation on December 13, 2019, were identified as SARS-CoV-2 positive (Basavaraju et al., 2020). It takes more than one week for humans to produce IgG after infection (Long et al., 2020; Xiang et al., 2020), indicating SARS-COV-2 might have already spread in the world earlier than it appeared in China.

The common symptoms of COVID-19 patients include fever, cough, breathlessness, and dyspnea. In more severe cases, SARS-COV-2 infection can lead to pneumonia, kidney failure, and even death (Berlin et al., 2020; Gandhi et al., 2020). The coronavirus family contains four genera: αCoV, βCoV, γCoV, and δCoV. Mammalian coronaviruses are mainly α CoV and βCoV, which can infect a variety of animals including pigs, dogs, cats, mice, cattle, and horses. Avian coronavirus is mainly derived from γ and δ coronaviruses, causing a variety of birds to get the disease. Currently, seven members of the coronavirus family are pathogenic to human beings (Woo et al., 2009). In addition to the three high pathogenic coronaviruses (SARS-CoV, MERS-CoV, and SARS-CoV-2), the other four human coronaviruses (HCoV-229E, HCoV-OC43, HCoV-NL63, and HCoV-HKU1) usually cause mild-to-moderate upper respiratory diseases in people (Fung and Liu, 2019). Several structural proteins are anchored on the membrane surface of coronavirus, mainly spike (S), nucleocapsid (N), membrane (M), and envelope (E) proteins (Malik, 2020). S protein is a key antigen out of the virus membrane that stimulates the host to produce a significant number of neutralizing antibodies (NAbs). The S protein contains S1 and S2 subunits and forms a large trimer (20 nm in length), the receptor binding domain (RBD) on the S1 subunit can bind to human ACE2 protein on the surface of epithelial cells, while S2 mediates the subsequent membrane fusion that allows the virus to enter the host cytoplasm. M glycoprotein is the most abundant component in coronaviruses. To shape the membranous virions and integrate necessary components into nascent virions, the binding of N protein with viral RNA can form spiral N protein, which is mainly responsible for wrapping and assembling viral genomes, maintaining the stability of the viral structure. E protein participates in viral assembly by forming ion channels on the viral cell membrane (Weiss and Leibowitz, 2011; Malik, 2020).

## Principles of Serological Detection: Overview

When a virus invades the human body and releases virus antigens into the bloodstream, the human immune system is then triggered to continuously produce a large number of specific antibodies (IgM/IgA/IgG) that are more concentrated than the antigenic protein and last for a long time (Reading and Dimmock, 2007; Combadiere, 2020; Yu et al., 2020). Therefore, compared to viral antigens, researchers prefer to use serological antibodies as diagnostic targets to develop faster, easier, and more sensitive serological tests (Espejo et al., 2020). Based on the immunology principle, detecting virus-specific antibodies showed more accurate results than detecting total immunoglobulin (Ig). Therefore, various studies have selected S or N protein–specific antibodies (IgM/IgA/IgG) as SARS-CoV-2 diagnosis targets (Li and Li, 2020; Mekonnen et al., 2020). Furthermore, some researchers even chose the S1 subunit or the RBD (receptor binding domain) as an antigen to improve the specificity of detection (Chen et al., 2020; Khan et al., 2020; Okba et al., 2020). For these different types of antibodies, IgM and IgA are produced by host immune cells at an early stage of infection while IgG at the late stage (Woo et al., 2004; Lam et al., 2020; Ma et al., 2020). Vashist et al. reported that IgA was positive in the serum 5°days after infection while IgM after 10–30°days and IgG after 20–90°days (Vashist, 2020). Therefore, the effectiveness of the COVID-19 serological test at an early stage of viral infection is not satisfied. A study has shown that only 50% of patients with SARS-CoV-2 infection had IgM and IgG positive in their serum one week after the onset of symptoms (Wolfel et al., 2020). However, the serum antibody concentrations would increase significantly over time, while the viral load would gradually decrease (Guo et al., 2020; Xiang et al., 2020). This means that serological detection is more effective than RT-PCR at the middle–late stage of infection. Moreover, serological detection results can reflect the severity of the patient’s symptoms, while RT-PCR results can hardly do. Yu et al. showed that IgM and IgG levels were significantly higher in severe COVID-19 patients than in mild-to-moderate patients (Yu et al., 2020). Currently, most serological test kits use specific IgM and IgG as detection targets (Ward et al., 2020). As IgA concentrations are higher than those of IgM and IgG in the early stages of infection (Vashist, 2020), some studies have proposed detection methods for IgA. Moreover, it is believed that the combined detection of multiple antibodies is helpful to improve the diagnostic accuracy of COVID-19 (Ma et al., 2020; Su et al., 2020).

## Serological Tests for the COVID-19 Diagnosis

As mentioned above, most serological detection methods rely on the principle of antigen–antibody–specific binding by detecting antibody levels in the human serum. The main serological detection methods include the chemiluminescence immunoassay (CLIA), enzyme-linked immunosorbent assay (ELISA), lateral flow immunoassay (LFIA), and immunofluorescence assay (IFA) (Ejazi et al., 2021; Mekonnen et al., 2020). These methods all have their own advantages and disadvantages, based on detection efficiency, system cost, the convenience of operation, and diagnosis restrictions (Kubina and Dziedzic, 2020), which will be fully discussed in this review.

### Enzyme-Linked Immunosorbent Assay

ELISA is the most commonly used classical serological test, with an average detection time between 2 and 8 h (Mekonnen et al., 2020). It was invented in the 1960s and is widely used from the 1970s till now (Lequin, 2005). According to the properties of detected target proteins and detection strategies, the ELISA can be divided into the sandwich ELISA, indirect ELISA, double antibody ELISA, competitive ELISA, blocking ELISA, and other different types (Butler, 2000). The indirect ELISA method is most commonly used in COVID-19 serological diagnosis (Mekonnen et al., 2020). The basic process of this method is to coat virus protein (N, S), S protein subunit (S1), or protein domain (RBD) on the solid phase carrier that binds with serum antibody and enzyme-linked antibodies to produce a chromogenic reaction (Lin, 2015). In addition to serum testing, Varadhachary et al. have developed a small automated ELISA device for detecting SARS-CoV-2–specific IgA levels in human saliva that can be used for the standard ELISA in less than 15 min (Varadhachary et al., 2020). Waleed established an ELISA test to detect antibodies against the SARS-CoV-2 S protein and S1/S2 subunit in the serum and compared their performance. They found the full-length S protein showed the strongest reactivity to IgG, while S1 showed the highest specificity (Mahallawi, 2021). Isho et al. designed an ELISA test to detect S protein–specific and RBD-specific IgG/IgA/IgM in patients’ serum (Isho et al., 2020). At present, there are many commercial SARS-CoV-2 detection kits by targeting different types of antigens and antibodies on the market (Lassaunière et al., 2020; Whitman et al., 2020), and the effectiveness of these kits will be evaluated in this review. Because the operation process of ELISA is comparably complicated (Sidiq et al., 2020), some manufacturers completed the encapsulated and closed steps beforehand and tried to merge the subsequent detection steps to shorten the procedure time (Manalac et al., 2020).

### Chemiluminescence Immunoassay

The principle of the CLIA is similar to that of the ELISA taking advantage of the high binding affinity between viral antigens and host antibodies, but the difference is the CLIA uses a chemical reaction to produce a glowing chemical probe to detect a positive signal (Weeks et al., 1986; Lin et al., 2019). Typically, CLIA results are obtained in 0.5–2 h (Vashist, 2020; Weeks et al., 1986). Like the ELISA, the CLIA is a high-throughput assay with higher accuracy and a low signal-to-noise ratio (Padoan et al., 2020; Tan et al., 2020). Taking S protein–specific IgG, for example, S-conjugated magnetic beads are co-incubated with serum samples and anti-human enzyme-linked antibodies to produce chemiluminescence. Currently, the detection steps after the magnetic bead–conjugated antigen are usually performed by the chemical immuno-luminescence analyzer, which greatly shortens detection time but increases the reliance on large detection instruments (Cai et al., 2020; Lin et al., 2020). Cai et al. used the combined detection of IgM and IgG antibodies and improved the performance of CLIA detection compared to the single antibody detection. They observed a positive rate of 81.52% for IgM and IgG tests, higher than that of the IgG test (57.2%) or IgM test (71.4%) alone (Cai et al., 2020). They used a magnetic chemiluminescent enzyme immunoassay (MCLIA) to detect antibodies against the N and S antigens of SARS-CoV-2 (Cai et al., 2020). Similarly, Ma et al. used the CLIA method to detect RBD-specific IgA, IgM, and IgG in the blood of patients with SARS-CoV-2 infection, with a sensitivity of 98.6, 96.8, and 96.8%, respectively, and a specificity of 98.1, 92.3, and 99.8%, respectively. On combined detection of the three antibodies, the sensitivity and specificity increased to 99.5 and 100%, respectively (Ma et al., 2020). Currently, the accuracy of CLIA test results is often higher than that of other methods’ results.

### Lateral Flow Immunoassay

**FIGURE 1:**
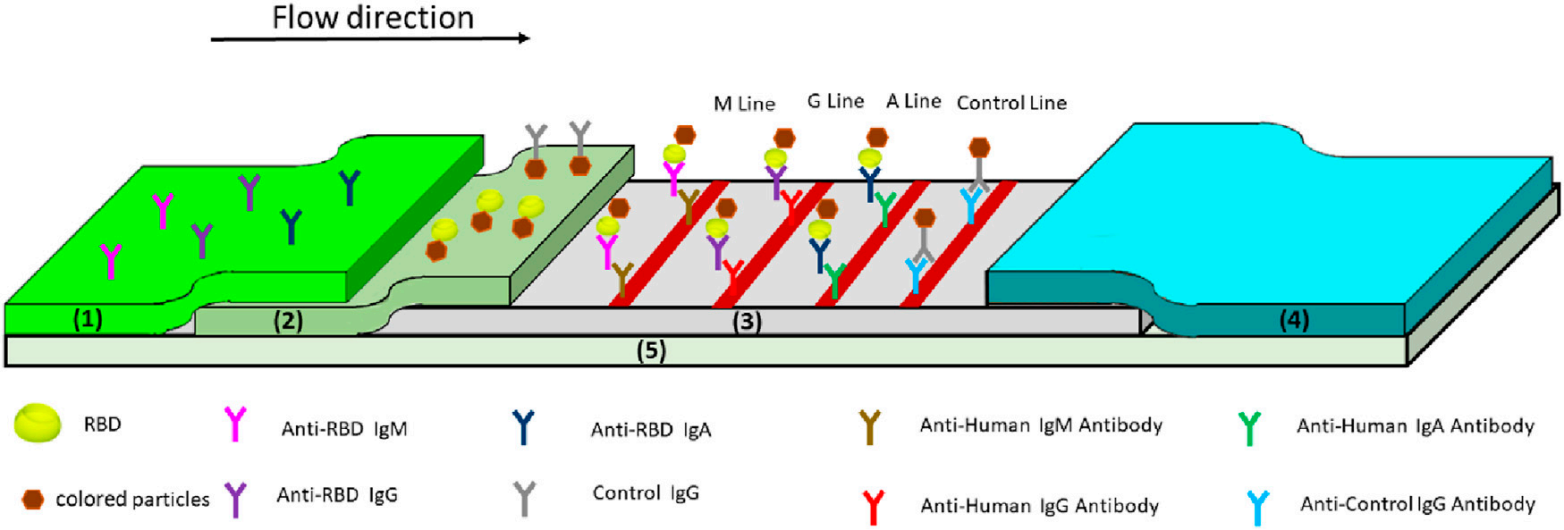
Schematic of the lateral flow immunoassay for testing anti-SARS-COV-2 antibodies (Li, Yi et al., 2020). **(1)** Sample pad, **(2)** conjugate release pad, **(3)** membrane with immobilized antibodies, **(4)** adsorbent pad, and **(5)** adhesive pad.

Lateral flow immunoassay (LFIA) is a low-cost, simple, fast, and portable test. It is widely used in biomedical, agricultural, food, and environmental sciences. For example, pregnancy test kits in the health field are successful applications of this technology (Koczula and Gallotta, 2016). In 1988, the Unilever subsidiary invented the first commercial home pregnancy test based on a transverse flow test (Ernst et al., 2021). Actually, the LFIA is a paper-based detection and analysis platform. This instant diagnostic method only needs 3–30 min to complete all processes as well as a small amount of sample load (Carrio et al., 2015; Moreno et al., 2017). A variety of biological samples can be used for LFIA detection, including serum, plasma, whole blood, urine, saliva, tears, and other liquids (Magambo et al., 2014; Schramm et al., 2015; Ang et al., 2016). As shown in Figure 1, typical LFIA equipment usually consists of five elements: sample pad, conjugate release pad, membrane with immobilized antibodies, adsorbent pad, and adhesive pad (Koczula and Gallotta, 2016; Montesinos et al., 2020). By siphoning a liquid sample, fixed antibodies on the dipstick can interact with the target molecule (Anfossi et al., 2018; Bever et al., 2020). Most LFIA tests for COVID-19 diagnosis use colloidal gold labeled with SARS-CoV-2 antigen. After identifying the corresponding antibody, the SARS-CoV-2 antibody can capture virus antigen from serum or whole blood (Machado et al., 2020; Goudouris, 2021). Therefore, the LFIA has the potential to be used for large-scale serological and instantaneous COVID-19 diagnosis. The process of LFIA for COVID-19 antibody diagnosis is definitely simple. Taking S protein–specific IgG, for example, a drop of whole blood is added to the sample pad and then made to flow through a conjugate release pad containing S protein–specific IgG and control IgG (such as rabbit IgG) conjugated with colored or fluorescent particles. Accompanied by the solution flow, the colored particle–conjugated complexes bind to specific antibodies immobilized in the detection region (Deeks et al., 2020; Ghaffari et al., 2020; Nicol et al., 2020). Although the LFIA is currently the most convenient serological test for SARS-CoV-2, it is prone to give false-negative and false-positive results than other methods (La Rosa Fabian and Urquizo Briceno, 2020; Montesinos et al., 2020). Hu et al. found that EDTA-K2 could chelate with colloidal gold, and the chelated colloidal gold was adsorbed on a conjugate release pad, which significantly improved the sensitivity and specificity of the diagnosis (Hu et al., 2021). Stieber et al. reported an LFIA detection device using fluorescent nanoparticles as a signal for antibodies against SARS-CoV-2, showing 100% specificity and sensitivity by qualitative detection of the IgM, IgA, and IgG antibodies against SARS-CoV-2 (Stieber et al., 2020). The results of this study need further independent evaluation, but if more researchers can continue to improve the detection accuracy of the LFIA, this method will display greater commercial application value.

### Immunofluorescence Assay

The IFA and ELISA have similar detection strategies, except that the results of the IFA test are shown by fluorescence microscopy. The basic detection process of IFA is to fix SARS-CoV-2–infected animal cells (such as Vero cells) on the glass slide, incubated with the patient’s serum. After adding fluorescent protein–labeled goat anti-human secondary antibodies, results can be obtained under the fluorescence microscope (Kohmer et al., 2020). This process requires live SARS-CoV-2 virus in the cells, increasing the potential risk of infection. Moreover, the IFA method requires people to observe the fluorescence intensity of cells under microscopy, making the results subjective to a certain extent (Haveri et al., 2020; Goudouris, 2021). The IFA test is also more complicated, subjective, and time-consuming (Venter and Richter, 2020). Due to these disadvantages, the IFA test has not been widely used in the serological diagnosis of SARS-CoV-2 infection.

## Performance of Serological Tests

With the increasing severity of the COVID-19 epidemic, the need for the development of serological diagnostic technology has become more and more urgent. Governments all over the world are eagerly supporting the development and commercialization of serological tests for SARS-CoV-2. Since the beginning of 2020, a large number of serological tests and kits for SARS-CoV-2 from a variety of institutions have been reported. Because of this, an independent and objective evaluation of various COVID-19 serological tests is needed to ensure the effectiveness of these tests and provide some information for future studies. Sensitivity and specificity are two basic quantitative criteria for evaluating diagnostic methods (Mandrekar, 2010). Sensitivity refers to the ability of these tests to identify positive samples. The higher the sensitivity, the fewer the false-negative results. Specificity is the ability of these tests to identify negative samples, and the higher the specificity, the fewer the false-negative results (Li et al., 2020; Goudouris, 2021).

Over the past year, there have been an increasing number of independent studies on serological tests for SARS-CoV-2, mainly involving performance evaluation and improvement of testing methods. We reviewed as many relevant studies as possible and systematically analyzed their data. As shown in Table 1, we collected 68 test results, including 32 ELISA tests, 15 CLIA tests, 18 LFIA tests, and 3 IFA tests (some of them from the same study). The tests were classified by different methods: ELISA, LFIA, CLIA, or IFA. We carefully summarized sensitivity and specificity for antigen (N, S, S1, and RBD)-specific antibodies (IgG, IgM, IgA).

**TABLE 1:** Summary of previous studies on sensitivity and specificity of COVID-19 serological diagnosis.

Methods and studies	Antigens	IgM		IgG		IgM or IgG		IgA	
		Sensitivity (%)	Specificity (%)	Sensitivity (%)	Specificity (%)	Sensitivity (%)	Specificity (%)	Sensitivity (%)	Specificity (%)
ELISA									
Lassaunière et al. (2020)	S			65.0	96.0			90.0	93.0
Guo et al. (2020)	N	85.4		77.9				92.7	
Dogan et al. (2020)	N			99.1	94.9				
	RBD	100	90.9	100	99.3			94.3	87.9
Snoeck et al. (2020)	SARS-CoV-2			97.8	85.7			89.2	92.9
Varadhachary et al. (2020)	N, S1							92.0	97.0
Bailey et al. (2020)									
Kit 1	N			100	99.6				
Kit 2	S1			99.4	99.6				
Kit 3	S			97.6	99.3				
Kit 4	S1			94.4	99.6				
Kit 5	S1							100	92.5
Kit 6	S			85.7	100				
Bortz et al. (2020)	S			91.0	99.0			70.0	99.0
Iyer et al. (2020)	S	81.0	100	97.0	100			91.0	100
GeurtsvanKessel et al. (2020)									
Kit 1	RBD	99.0	90.0	99.0	81.0			94.0	97.0
Kit 2	S1								
Kit 3	S1								
Fujigaki et al. (2020)									
Kit 1	RBD	79.7	94.0	77.9	98.0			80.2	97.0
Kit 2	S1	76.8	99.0	72.1	97.0			79.1	94.0
Kit 3	N	63.8	88.0	84.3	79.0			80.2	82.0
La Rosa Fabian and Urquizo Briceno (2020)	S							92.3	65.4
Huynh et al. (2020)	S	100	95.5	100	96.7			100	97.6
	RBD	100	97.3	100	94.6			100	97.0
Tre-Hardy et al. (2021)	N	48.7	98.7	94.9	96.2			89.7	98.7
Nuccetelli et al. (2020)									
Kit 1	N	80.0	100	94.0	93.0			82.0	93.0
Kit 2	N	92.0	100	98.0	94.0			92.0	94.0
Mazzini et al. (2021)	RBD			85.7	98.1				
Van Elslande et al. (2020)									
Kit 1	S	39.2	91.3	62.1	98.1	65.4	90.3		
Kit 2	S	72.5	95.1	68.0	93.2	76.5	91.3		
Kit 3	S	65.4	100	62.8	99.0	65.4	99.0		
Kit 4	S	32.0	99.0	64.7	99.0	66.7	98.1		
Kit 5	N	69.3	95.1	61.4	99.0	69.3	95.2		
Kit 6	N	43.8	91.3	64.7	97.2	71.2	88.3		
Kit 7	N	56.2	93.2	71.2	90.3	79.1	85.4		
Jaaskelainen et al. (2020)	S1			33.3	91.9			61.5	73.0
Beavis et al. (2020)	S1			82.9	88.4			67.1	97.1
Sterlin et al. (2020)	S					91.0	98.0		
Pieri et al. (2020)									
Kit 1	N			33.3–80.0	98.5			50.0–100	92.5
Kit 2	N	88.2	92.0	92.5	93.3				
Liu, Liu, Kou et al. (2020)	N	68.2	100	70.1	100	80.4	100		
	S	77.1	100	74.3	100	82.2	100		
Adams et al. (2020)	S	70.0	100	85.0	100	85.0	100		
Whitman et al. (2020)	S	56.9	96.9	73.8	88.8	75.4	87.5		
	N					72.3	95.0		
Liu, Liu, Zheng et al. (2020)	N					83.0	96.6		
Freeman et al. (2020)	S					96.0	99.3		
Zhao et al. (2020)	N, S	82.7	98.6	64.7	99.0	99.3	99.1		
Lou et al. (2020)	S	92.5	100	88.8	99.0	97.5	99.1		
Zhong et al. (2020)	S	97.9	99.7	97.9	99.7				
Xiang et al. (2020)	N	77.3	100	83.3	95.0				
Perera et al. (2020)	RBD	78.7	100	72.3	98.6				
Nguyen et al. (2020)									
Kit 1	N					92.2	99.6		
Kit 2	S					90.0	100		
Kit 3	S					92.5	100		
Zava and Zava (2021)									
Kit 1	N			90.0	100				
Kit 2	N			98.4	99.8				
Kohmer et al. (2020)				70.6	83.3				
CLIA									
Ma et al. (2020)	RBD	96.8	92.3	96.8	99.8	98.6	100	99.6	98.1
Marklund et al. (2020)	RBD	37.5	100	91.7	100			45.8	100
GeurtsvanKessel et al. (2020)	S					90.0	81.0		
Pieri et al. (2020)									
Kit 1	N					65.5–100	99.8		
Kit 2	N, S	78.6	97.5	91.2	97.3				
Manalac et al. (2020)	N		97.9	99.6					
Lou et al. (2020)	S	86.3	99.3						
Zhong et al. (2020)	S	97.9	95.3	95.7	96.7				
Lin et al. (2020)	RBD	81.2	82.3	97.5	82.3	80.0	91.1		
Cai et al. (2020)	S	57.2	100	71.4	100	81.5	100		
Infantino et al. (2020)	N, S	73.3	92.2	76.7	100				
Jin et al. (2020)	N, S	48.1	100	88.9	90.9				
Xie et al. (2020)	N, E	93.8	85.0	100	100				
Yangchun (2020)	S	70.2	96.2	96.1	92.4				
Qian et al. (2020)	N, S	85.9	98.1	96.6	98.1				
Nguyen et al. (2020)									
Kit 1	S			90.0	100				
Kit 2	S					100	100		
Kit 3	N					100	99.8		
LFIA									
Lassaunière et al. (2020)									
Kit 1	Unknown					90.0	100		
Kit 2	Unknown					90.0	100		
Kit 3	Unknown					93.3	100		
Kit 4	Unknown					83.3	100		
Kit 5	Unknown					80.0	80.0		
GeurtsvanKessel et al. (2020)									
Kit 1	S, N					99.0	89.0		
Kit 2	N					85.0	90.0		
Kit 3	S, N					88.0	100.0		
La Rosa Fabian and Urquizo Briceno (2020)	S	66.7	69.2						
Nuccetelli et al. (2020)									
Kit 1	N			71.0	94.0	80.0	96.0		
Kit 2	N			84.0	94.0	71.0	96.0		
Van Elslande et al. (2020)	N	55.6	96.1	92.1	90.3	70.0	85.4		
Whitman et al. (2020)									
Kit 1	N	62.7	87.9	56.3	96.3	65.9	86.9		
Kit 2	N	72.2	97.1	63.5	98.1	75.4	95.2		
Kit 3	N	68.5	90.7	67.7	91.6	52.4	89.7		
Kit 4	N	73.4	84.3	53.9	99.1	74.2	84.3		
Kit 5	S	29.2	96.3	54.9	100	58.4	96.3		
Kit 6	S	70.1	98.1	54.3	99.1	71.7	97.2		
Kit 7	S	48.8	100	57.5	100	50.4	100		
Kit 8	S	57.8	98.1	57.0	98.1	46.9	98.1		
Kit 9	S	66.4	94.9	64.7	96.0	66.4	94.9		
Kit 10	N					70.2	99.1		
Lou et al. (2020)	S	88.8	98.1	86.3	99.5	97.5	95.2		
Li et al. (2020)	S	82.6	91.4	70.5	94.1	88.7	90.6		
Pérez-García et al. (2020)	N	21.8	100	41.8	100	47.3	100		
Imai et al. (2020)	N	43.2	97.9	14.4	100	43.2	97.9		
Ying et al. (2020)	S	37.8	93.3	83.3	92.1	85.6	91.0		
Cassaniti et al. (2020)	S	83.3	100	80.0	100	83.3	100		
Hoffman et al. (2020)	N, S	69.0	100	93.1	99.2				
Chen et al. (2020)	N, S			100	91.7				
Zhang et al. (2020)	RBD					86.9	99.4		
VirgilioParadiso et al. (2020a)	RBD					30.7	89.2		
Nguyen et al. (2020)									
Kit 1	S	95.7	99.7	99.0	99.4	99.0	99.0		
Kit 2	S					93.8	96.0		
Kit 3	N	77.4		87.1		93.5	94.4		
Kohmer et al. (2020)				62.5	100				
IFA									
Jia (VirgilioParadiso et al., 2020b)		72.0	39.0	71.0	52.0	87.5	72.7		
Kohmer et al. (2020)				76.5	86.4				
Edouard et al. (2021)				41.0	93.0				

**FIGURE 2:**
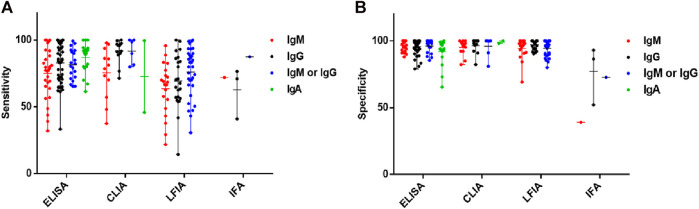
Effectiveness of different serological diagnosis tests for anti-SARS-COV-2 antibodies. Sensitivity **(A)** and specificity **(B)** of ELISA, CLIA, LFIA, and IFA tests for anti-SARS-COV-2 IgM, IgG, and IgA.

We compared the differences in diagnostic effects of different detection techniques or different detection target antibodies (Figure 2). In a word, the ELISA was less sensitive than the CLIA but more sensitive than the LFIA in COVID-19 diagnosis, meaning that the CLIA test is more effective for the diagnosis of early infected patients with low antibody concentrations. The specificity of the CLIA and LFIA was similar, but the overall specificity of the CLIA was slightly higher than that of the other two tests (Figure 2B). Thus, the CLIA performed best overall in COVID-19 diagnosis, while the LFIA technology increased its accuracy. In addition, comparing the results of different targeted antibodies, we found the combination of IgM and IgG detection could significantly improve the sensitivity of the above three detection methods (Figure 2A). Moreover, the sensitivity and specificity of the ELISA for IgA detection are very high, which means that IgA may be widely used as an important indicator. The number of IFA tests was too small for statistical analysis. Besides, we compared the merits and drawbacks of COVID-19 serological diagnosis methods according to several evaluation criteria including sensitivity, specificity, cost, simplicity, and security (Table 2).

**TABLE 2:** Comparison and evaluation of COVID-19 serological diagnosis methods.

	ELISA	CLIA	LFIA	IFA
Sensitivity	Medium	High	Low	Medium
Specificity	Medium	High	Medium	Low
Cost	Medium	High	Low	Medium
Simplicity	Medium	Relatively simple	Simple	Complicated
Convenience	Lab/hospital	Lab/hospital	Portable	Lab
Security	Relatively safe	Safe	Safe	Dangerous

## Discussion

COVID-19 has now become the most prevalent and widespread plague in the global human society. Due to the inability of most countries to control COVID-19, it is likely to continue to rage for a long time, and both developed and undeveloped countries are scrambling to stockpile vaccines. SARS-CoV-2 has been reported in multiple countries with different mutations, which increased infectiousness and resistance, resulting in cross-infection of different SARS-CoV-2 mutant strains in some patients (Gong et al., 2020; Sun et al., 2020; Leung et al., 2021). It is difficult to ensure the existing vaccines remain effective against reported or unknown mutant strains (Veljkovic et al., 2020; Lauring and Hodcroft, 2021; Veljkovic et al., 2021). In addition, virus antibody dynamics studies have shown that serum SARS-CoV-2–specific antibodies levels would rapidly drop 50% in 5°months after infection (Conte, 2020; Du et al., 2020; Woo et al., 2004). Based on the fact that SARS-CoV-1 patients’ IgG can last 8–24°months after the onset of symptoms, the COVID-19 patients may encounter the secondary infection when their antibodies disappear (Liu et al., 2006; Woo et al., 2004; Wu et al., 2007).

These discouraging findings suggest that SARS-CoV-2 may not be eliminated altogether but instead become a seasonal virus, like the flu virus (Neher et al., 2020; Poole, 2020). Therefore, the development and popularization of various COVID-19 diagnostic technologies are needed for controlling the epidemic. There are two main diagnostic techniques: molecular tests and serological tests. Initially, most of the attention focused on the SARS-CoV-2 molecular assay, which can detect virus-specific RNA molecules circulating in the host with high precision. The gold standard for molecular testing is based on reverse transcription polymerase chain reaction (RT-PCR), a routine confirmation test conducted by the World Health Organization (WHO). At an early stage of SARS-CoV-2 infection, the viral load in the human body is considerably high, while the specific antibodies appear several days later and the concentration is low at that time. Therefore, the accuracy of RT-PCR for COVID-19 patients at an early stage of infection is reliable. However, Li et al. reported that the specific antibodies of SARS-CoV-2 could still be detected in the serum of PCR-negative patients. With the initiation of human immune response and the reduction of viral load, the accuracy of nucleic acid detection began to decrease, and the diagnostic validity of serological detection began to exceed that of the nucleic acid assay (Guo et al., 2020). Serological tests can not only replace nucleic acid tests and reduce the proportion of false-negative diagnoses but also help retrospectively assess the incidence and phase of an outbreak in an area.

The development of effective and reliable serological detection methods plays a vital role in monitoring the abundance and neutralization efficiency of antibodies in infected patients, evaluating and predicting the severity of symptoms of patients, and quantifying the quality of immune response to newborn vaccines. In addition, a variety of serological tests are cost-effective, convenient, and efficient than RT-PCR detection for commercial application. Here, we reviewed the latest knowledge on the composition and function of SARS-CoV-2 and the humoral immune response it causes, as well as on the current popular serological detection techniques, including the ELISA, CLIA, LFIA, and IFA. We present the fundamentals of these commercial and laboratory-based detection techniques and the advantages and limitations of application in COVID-19 diagnosis. In addition, we summarized published reports that have evaluated various serological tests, analyzing and comparing the effectiveness of these tests in the diagnosis of COVID-19. In terms of serological diagnosis techniques, although the ELISA and CLIA have shown relatively prominent accuracy, they are still limited to laboratories and clinics due to the complex testing process, high cost, and reliance on sophisticated detection instruments. The experimental conditions of the IFA are more hazardous, and the test results are not easy to be quantified. The LFIA test is more prone to give false-positive and false-negative results than other test technologies, but it has the advantages of convenience, simplicity, low cost, and the potential for large-scale screening.

Given our collection of reported studies, the performance of these serological tests varies widely, with some tests falling far short of the sensitivity and effectiveness criteria proposed by the FDA. In particular, some serological tests showed the sensitivity at an early stage of infection is much lower than that at a late stage of infection. Moreover, the sensitivity can approach 90% only for patients who have been infected more than 15°days, implying that enhanced diagnosis accuracy is still necessary (Fujigaki et al., 2020; Wolf et al., 2020). Already, some commercial tests on the market have been recalled due to substandard performance. At the same time, among these different serological detection techniques, a considerable number of studies have reported good detection effects. Due to the limitations of the researchers’ environment and scientific study conditions, these independent investigations typically detected just 10–50 patient samples, which is insufficient for validation. Therefore, good performance based on a large number of samples will contribute to the development and improvement of serological test technology.

## Concluding Remarks

In this review, we discussed the most prevalent serological diagnostic approaches for SARS-CoV-2–specific antibodies, compared their merits and drawbacks, and evaluated their efficacy in diagnosing COVID-19. The potential and significance of serological tests in the diagnosis of COVID-19 are widely understood. More and more serological test technologies and commercial serological tests are being developed. Although the results of serological tests vary greatly, some of them display excellent performance. With technological innovation and rigorous regulatory evaluation, the accuracy and effectiveness of serological tests will continue to help prevent the SARS-CoV-2 infection.
